# Synchronous Clear-Cell Renal Cell Carcinoma and Ipsilateral Renal Calculus in the Same Kidney: A Case Report and Review of the Literature

**DOI:** 10.7759/cureus.114428

**Published:** 2026-08-12

**Authors:** Ashwin Vinod, Rajendra B Nerli, Shridhar Ghagane, Neeraj Dixit

**Affiliations:** 1 Urology, KLE Academy of Higher Education and Research, Jawaharlal Nehru Medical College, Belagavi, IND; 2 Urology, KLE Centenary Charitable Hospital, Belagavi, IND

**Keywords:** nephron-sparing surgery, partial nephrectomy, radical nephrectomy, renal cell carcinoma, renal stone, urolithiasis

## Abstract

Urolithiasis is a common urinary tract disorder in adults, yet the synchronous occurrence of renal cell carcinoma (RCC) and a calculus within the same kidney is seldom reported. We describe a 63-year-old man who presented with right loin pain and was found on evaluation to have RCC with an ipsilateral renal stone. Ultrasonography demonstrated a 3 cm right renal pelvic calculus, and contrast-enhanced computed tomography further revealed a 5.0 × 4.7 × 4.0 cm ipsilateral renal mass. He underwent open partial nephrectomy with concurrent pyelolithotomy, and histopathology confirmed clear-cell RCC with free surgical margins. Synchronous renal calculi and RCC are rare. Management is guided by the clinical stage of the tumor, which also determines the oncological outcome. The two conditions nonetheless share recognized risk factors, such as obesity and hypertension, and a thorough cross-sectional evaluation of patients with renal stones is warranted so that an incidental mass is not overlooked.

## Introduction

Urolithiasis is among the most prevalent disorders of the urinary tract, with a lifetime prevalence approaching 1 in 10 adults and a rising global burden that parallels the worldwide increase in obesity, type 2 diabetes, and metabolic syndrome [1,2]. Stone disease shows a male predominance and an incidence that increases steadily with age, mirroring the epidemiological profile of many urological malignancies [3,4].

Renal cell carcinoma (RCC) accounts for the majority of primary renal parenchymal malignancies, while urothelial (transitional cell) carcinoma predominates in the collecting system. The concurrent presence of a calculus and a solid renal mass within the same kidney is distinctly uncommon, and the mechanistic overlap between the two conditions remains only partly understood. Long-standing calculi sustain a milieu of mechanical irritation, recurrent infection, and chronic inflammation, a state that has been repeatedly implicated in urothelial proliferation and malignant transformation [5,6]. Whether these same influences meaningfully drive parenchymal rather than urothelial carcinogenesis is a question that current evidence answers only incompletely.

Beyond a shared inflammatory hypothesis, stone disease and RCC share a common set of predisposing factors, including obesity, hypertension, and diabetes, while additional exposures such as long-term analgesic use are independently recognized in renal carcinogenesis, together complicating any attempt to disentangle causation from confounding [7,8]. Against this background, the synchronous discovery of a renal mass in a patient investigated for stone-related loin pain carries practical importance: it can alter the surgical plan, mandate oncological follow-up, and reframe an apparently benign presentation. We describe a 63-year-old man in whom evaluation of a renal calculus led to the incidental detection of an ipsilateral clear-cell RCC, and we place the case within the contemporary literature on the stone-cancer relationship.

## Case presentation

**Figure 1:**
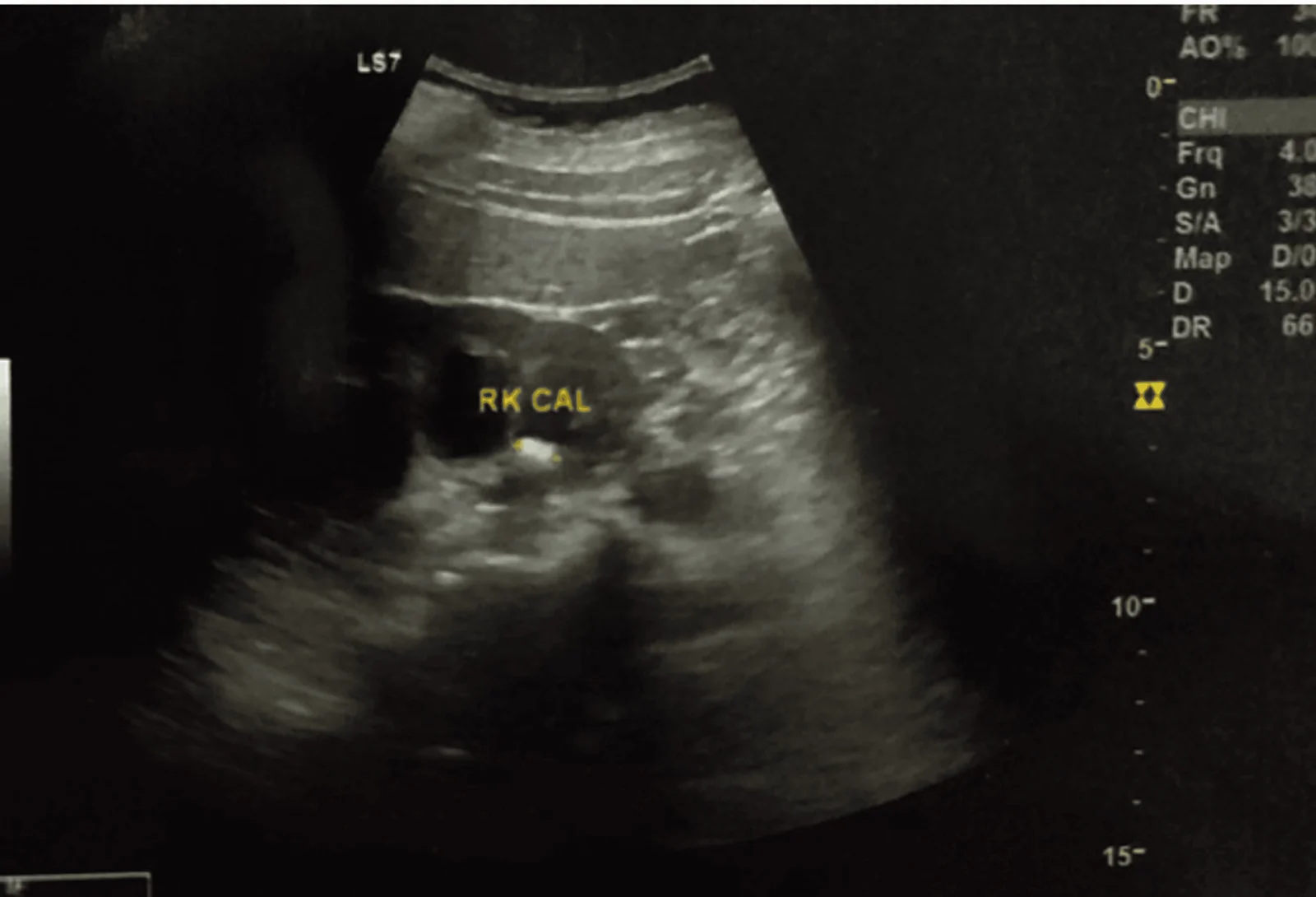
Ultrasonography showing 3 cm right renal pelvic calculus.

A 63-year-old man presented with right loin pain. He was mildly obese (74 kg, body mass index 28 kg/m^2^) and had been on antihypertensive therapy for 24 years. Initial abdominal ultrasonography performed by a family physician demonstrated a 3 cm right renal pelvic calculus (Figure 1).

**Figure 2:**
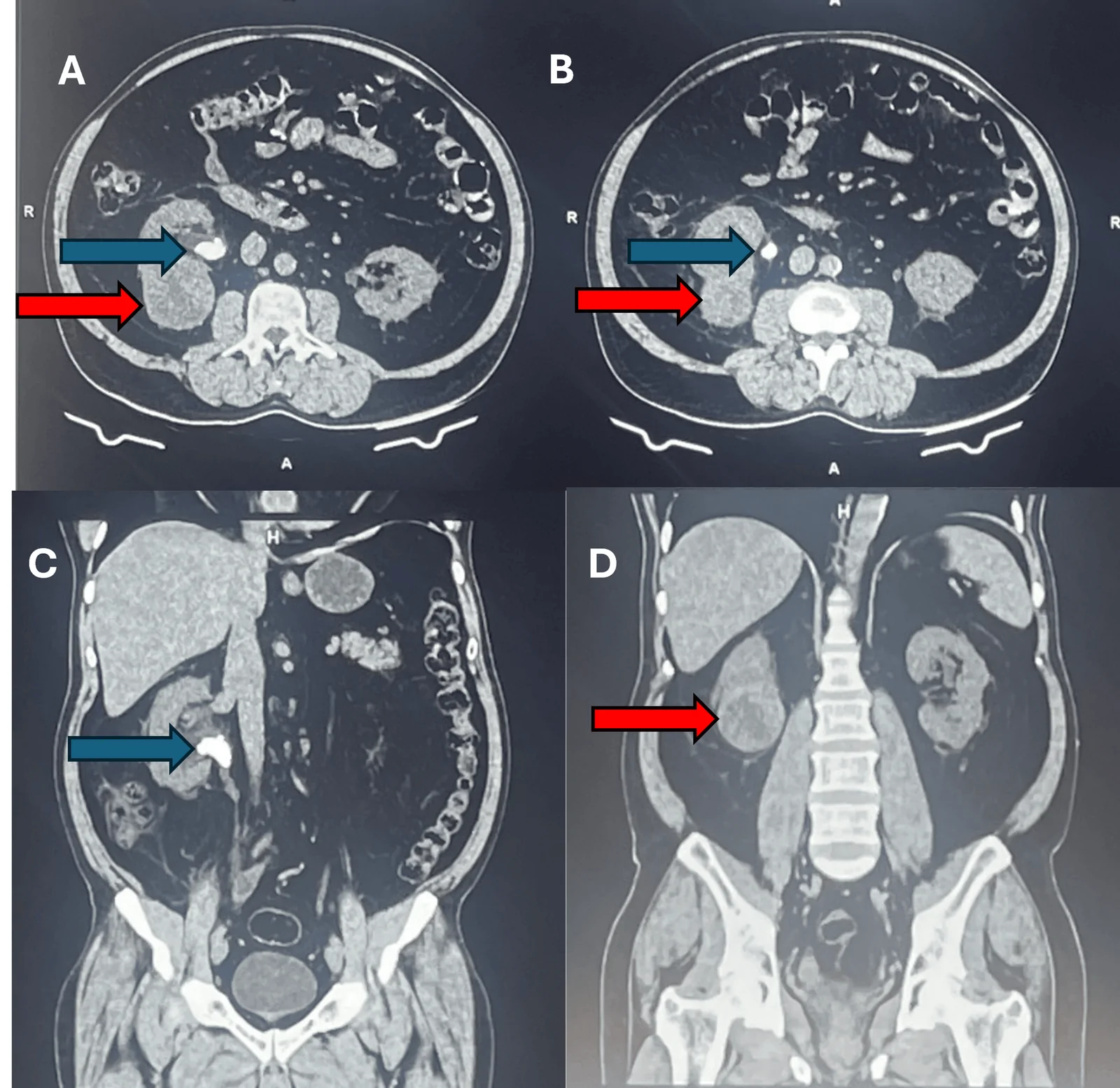
Noncontrast computed tomography of the abdomen showing right renal calculus and renal mass. (A, B) Axial sections. (C,D) Coronal sections. The blue arrow indicates calculus measuring 2.5 x 1.2 cm in the right renal pelvis extending into the right ureter. The orange arrow indicates a well-defined soft tissue mass in the lower pole of the right kidney.

**Figure 3:**
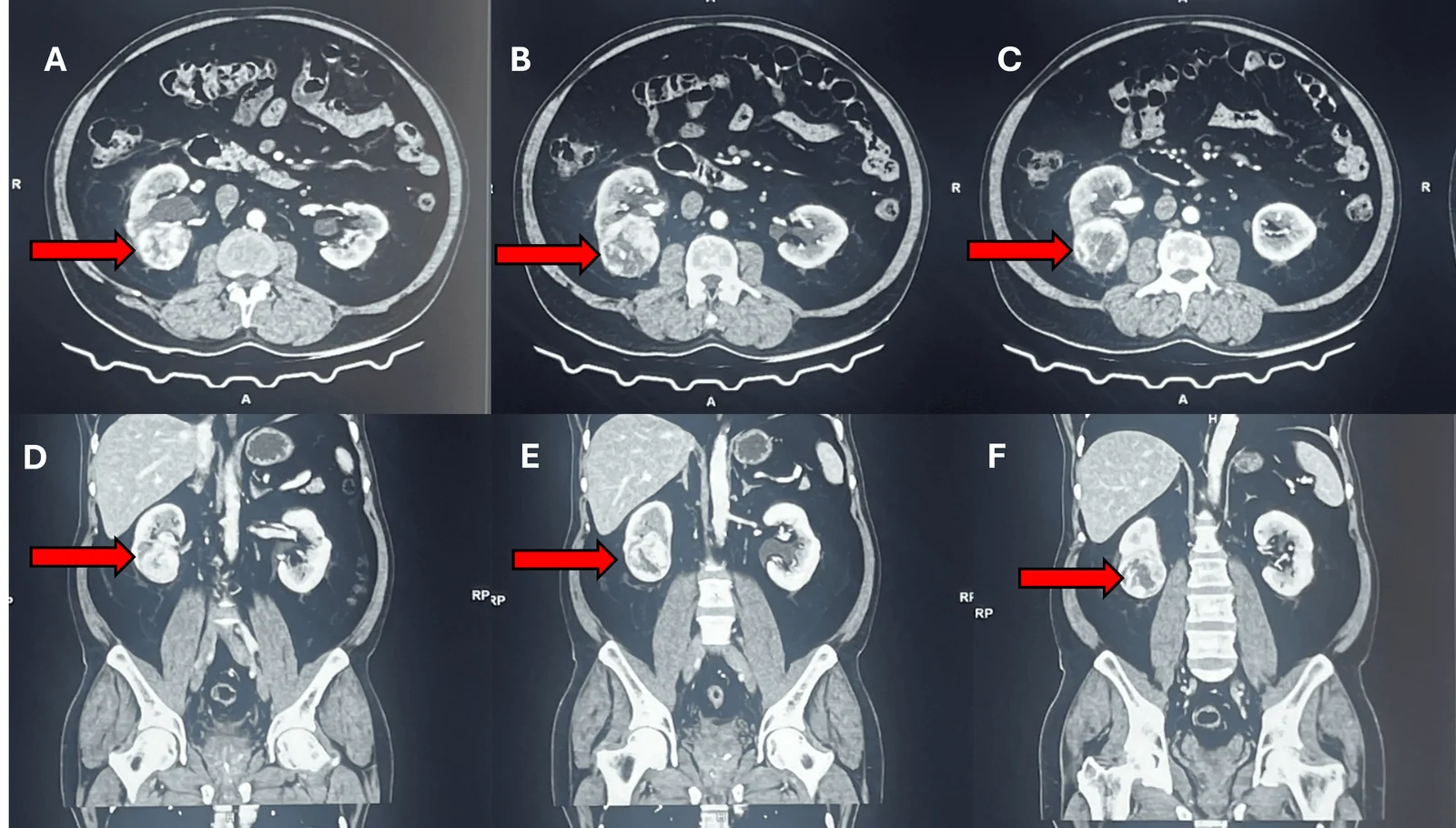
Contrast-enhanced computed tomography of the abdomen showing renal mass. (A, B, C) Axial section. (D, E, F) Coronal sections. Orange arrows indicate a well-defined, heterogeneously enhancing soft-tissue density mass lesion measuring 5.0 x 4.7 x 4.0 cm in the lower pole of the right kidney, along the postero-inferior aspect, with a predominantly exophytic component and no evidence of calcification or fat density lesions.

Contrast-enhanced computed tomography of the abdomen confirmed the calculus (Figure 2) and additionally revealed a 5.0 x 4.7 x 4.0 cm renal mass situated on the postero-inferior aspect of the right kidney (Figure 3). The lesion was well defined and heterogeneously enhancing, without calcification. It was predominantly exophytic, with a minimum distance of 7 mm from the pelvicalyceal system, features favorable for a nephron-sparing approach. 

**Figure 4:**
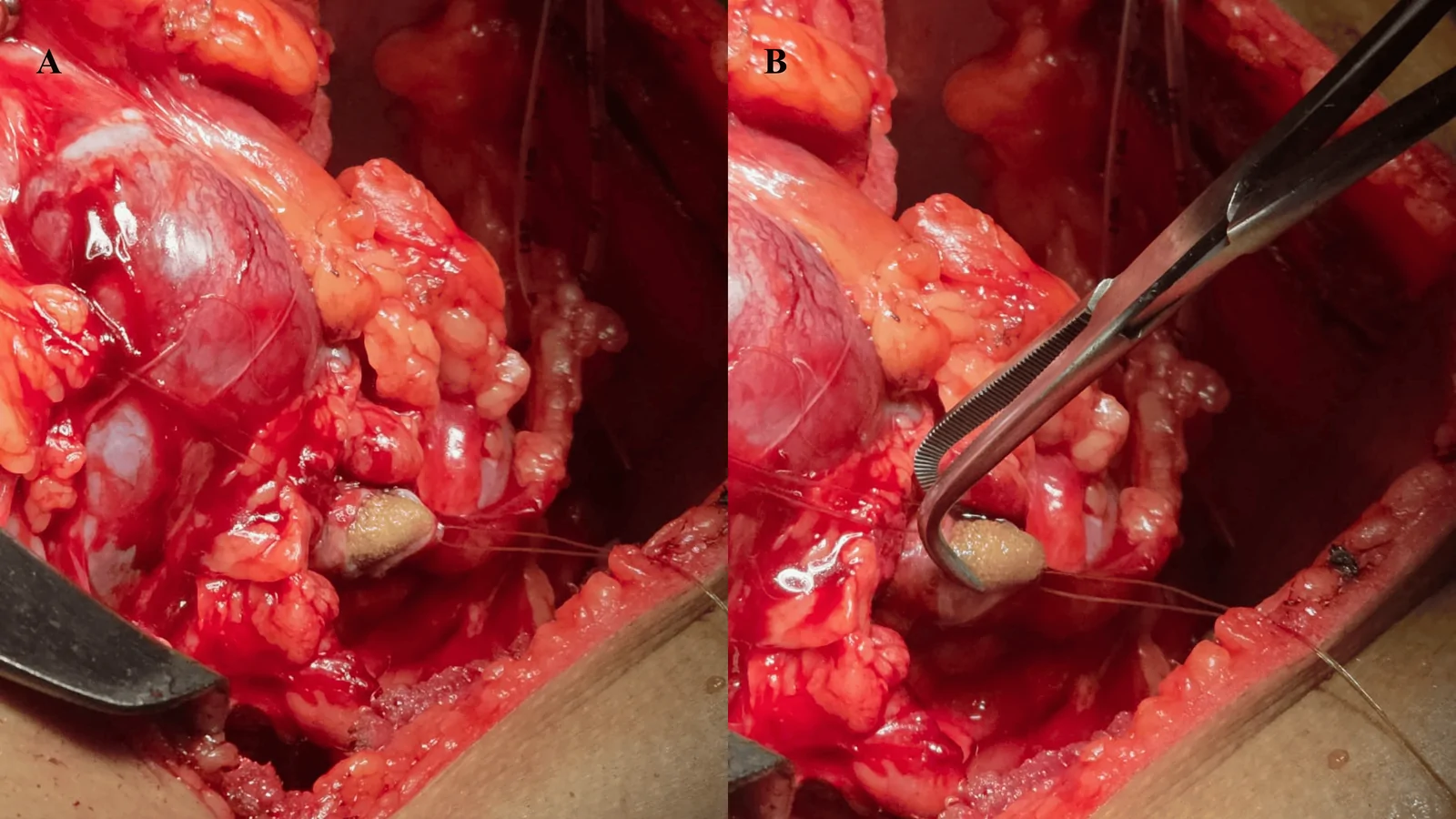
Intraoperative photographs showing removal of the right renal calculus. (A) Pyelolithotomy and visualization of calculus. (B) Calculus removed in toto.

**Figure 5:**
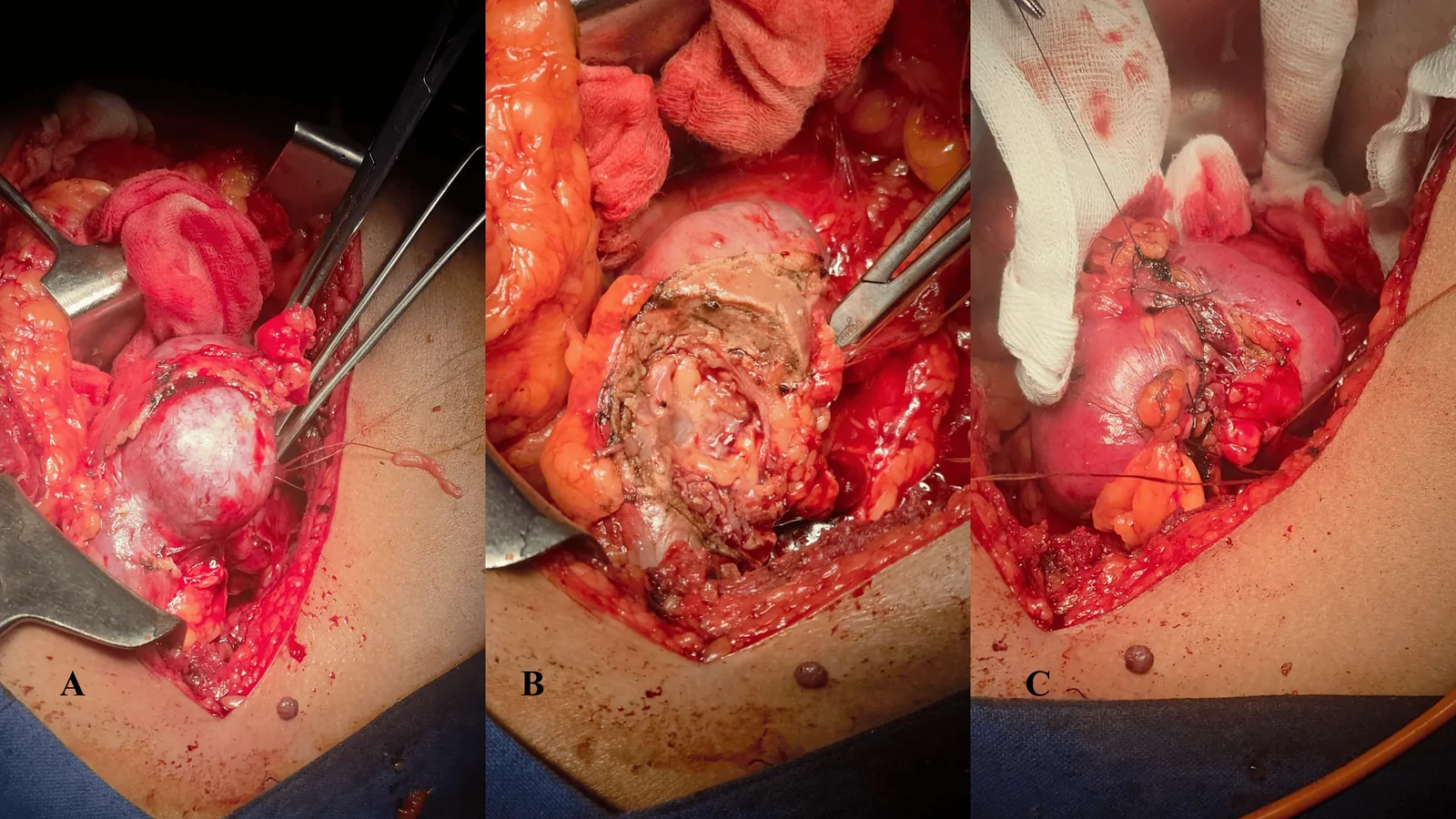
Intraoperative photographs showing renal cell caricoma. (A) Right lower-pole renal cell carcinoma identified and margins marked. (B) Right partial nephrectomy and removal of the mass in toto. (C) Closure of the parenchymal defect.

The patient was counseled for surgical exploration with partial nephrectomy and concurrent pyelolithotomy. The planned excision was carried out; both the calculus and the tumor were removed during the same procedure (Figures 4, 5). Recovery was uneventful, with no intraoperative or immediate postoperative complications. Histopathological examination confirmed clear-cell RCC with free surgical margins. 

## Discussion

Epidemiological association between calculi and renal malignancy

The possibility that nephrolithiasis predisposes to renal and upper-tract malignancy has been examined across case-control and cohort studies over three decades. Early hospital-based cohorts identified an excess of renal pelvis and ureteric cancer among stone formers [5], and a large population database from Taiwan subsequently confirmed an independent association between urinary calculi and later kidney cancer after adjustment for shared comorbidities [6].

The most influential quantitative synthesis is the meta-analysis by Cheungpasitporn and colleagues, which pooled data from case-control and cohort studies and reported an elevated risk of both renal parenchymal and upper-tract urothelial tumors in patients with a history of stones, with pooled risk ratios of approximately 1.76 for RCC and 2.14 for transitional cell carcinoma [9]. A more recent and larger synthesis incorporating 13 studies reaffirmed and slightly strengthened this signal, estimating a pooled kidney cancer risk ratio of roughly 2.36, albeit with substantial between-study heterogeneity [10].

Subtype-specific evidence: the Netherlands cohort study

The prospective Netherlands Cohort Study on diet and cancer, following more than 120,000 participants over two decades, provides the most granular subtype-resolved data available [11]. A history of kidney stones was associated with an increased overall RCC risk (hazard ratio (HR) 1.39, 95% confidence interval (CI) 1.05-1.84). Critically, the association was concentrated in the papillary subtype (HR 3.08, 95% CI 1.55-6.11), whereas clear-cell RCC showed no significant excess (HR 1.14, 95% CI 0.79-1.65). Upper-tract urothelial carcinoma risk was likewise elevated (HR 1.66, 95% CI 1.03-2.68).

Subtype-resolved data therefore suggest that the excess relative risk associated with a history of stones is most pronounced for papillary RCC, whereas no statistically significant excess was demonstrated for clear-cell tumors in that cohort. This should not be taken to mean that clear-cell RCC does not occur alongside calculi; clear-cell histology accounts for the majority of renal cell carcinomas and therefore remains the subtype most frequently encountered in stone formers in absolute terms, as in the present case. The distinction is one of etiological attribution rather than frequency: the failure to demonstrate a significant excess of clear-cell RCC derives from a single prospective cohort with wide confidence intervals, and absence of a demonstrated association is not evidence of its absence. Taken together, the available data make a direct lithogenic contribution to clear-cell carcinogenesis unlikely based on current evidence, and the coexistence in our patient is best interpreted as a synchronous presentation plausibly explained by shared metabolic risk factors, a nuance that adds to, rather than detracts from, the instructional value of the case [11]. A summary of the key studies on the stone-cancer association is presented in Table 1.

**Table 1:** Summary of key evidence on the association between urinary calculi and renal malignancy. RR: risk ratio; HR: hazard ratio; RCC: renal cell carcinoma; TCC: transitional cell carcinoma; UTUC: upper-tract urothelial carcinoma.

Study	Design/setting	Focus	Principal finding
Chow et al. (1997) [5]	Cohort, Sweden	Stone-forming hospital cohort followed for urinary-tract cancers	Excess risk of renal pelvis/ureter cancer among stone formers
Schlehofer et al. (1996) [12]	International case-control (RCC)	Role of medical and family history	Prior stone disease modestly raised RCC risk
Chung and Lin (2013) [6]	Population database, Taiwan	Urinary calculi vs. matched controls	Calculi independently associated with subsequent kidney cancer
Sun et al. (2013) [13]	Population cohort, Taiwan	Urinary-tract stone vs. subsequent urinary-tract cancer	Stones raised subsequent urinary-tract cancer risk
Cheungpasitporn et al. (2015) [9]	Meta-analysis (case-control + cohort)	Pooled RCC and TCC risk in stone formers	RCC RR 1.76 (1.24-2.49); TCC RR 2.14 (1.35-3.40)
Bhojani et al. (2025) [10]	Meta-analysis, 13 studies (5 cohort, 8 case-control)	Updated pooled estimate	Kidney cancer RR 2.36 (1.74-2.98)
van de Pol et al. (2019) [11]	Netherlands Cohort (n = 120,852)	20-year prospective, subtype-specific follow-up	Overall RCC HR 1.39; papillary RCC HR 3.08; clear-cell not raised; UTUC HR 1.66
Gupta and Kanwar (2021) [14]	Mechanistic review	Urinary dysbiosis and stone-cancer axis	Microbiome-driven inflammation proposed as a shared pathway

Proposed mechanisms of stone-associated carcinogenesis

Three complementary hypotheses recur in the literature. First, chronic mechanical trauma and recurrent infection maintain a sustained inflammatory response; cytokines and chemokines released by infiltrating inflammatory cells promote urothelial hyperproliferation, dysplasia, and, over time, malignant transformation, a sequence best characterized for squamous and transitional carcinomas of the collecting system [6,13]. Second, shared metabolic drivers such as obesity, insulin resistance, hypertension, and dyslipidemia independently elevate the risk of both stone formation and renal neoplasia, generating an epidemiological association without direct mechanistic linkage [15]. Third, and more recently, urinary and tissue dysbiosis has been proposed as a unifying axis, whereby an altered microbiome fosters both lithogenesis and a protumorigenic inflammatory microenvironment [14].

For parenchymal clear-cell RCC specifically, none of these pathways is firmly established, and the balance of subtype-specific evidence suggests that the stone-clear-cell relationship is more likely coincidental than causal. This distinction has direct implications for how synchronous presentations should be counseled and followed.

Interpretation of the present case

The present case illustrates a clinically instructive but epidemiologically infrequent scenario: the incidental detection of RCC during the workup of symptomatic stone disease. The value of the case lies less in the coexistence itself than in what it reveals about diagnostic vigilance and the limits of the stone-cancer hypothesis. From a diagnostic standpoint, the sequence of events is a reminder that ultrasonography, though an excellent first-line tool for calculi, may fail to detect a coexisting solid mass. Cross-sectional imaging was decisive here, revealing a well-defined, heterogeneously enhancing, predominantly exophytic lesion situated away from the pelvicalyceal system. This anatomy was favorable for nephron-sparing surgery, and open partial nephrectomy with concurrent pyelolithotomy allowed simultaneous removal of both the calculus and tumor with negative surgical margins, an approach consistent with current EAU and AUA recommendations, which favor partial nephrectomy for clinically localized cT1 renal masses, including selected cT1b tumors, wherever it is technically feasible and oncologically safe [16,17].

Interpreted against the literature, the case sits at an informative point of tension. Population data consistently associate a history of stones with a modestly increased RCC risk [9,11], yet the available subtype-resolved evidence points to a stronger relative association with papillary histology, with no significant excess demonstrated for clear-cell tumors in the single cohort addressing this question. The clear-cell histology in our patient therefore aligns with a coincidental synchronous presentation, most plausibly explained by shared metabolic risk factors; he was overweight and had a long history of hypertension, both recognized contributors to stone formation and renal neoplasia [15,18]. This interpretation tempers any tendency to infer direct causality from mere anatomical proximity.

Several evidence gaps remain pertinent. The direction and strength of any causal link between calculi and clear-cell RCC are unresolved; the influence of stone composition, laterality, and cumulative exposure to lithogenic solutes on renal cell biology is essentially unstudied; and the pronounced male predominance common to both conditions has not been mechanistically explained. Prospective work integrating stone analysis, metabolic phenotyping, and microbiome characterization would help separate shared-risk-factor confounding from genuine biological causation.

## Conclusions

Synchronous nephrolithiasis and renal cell carcinoma in the same kidney is rare. While stone disease is an epidemiologically recognized risk marker for renal malignancy, the strongest relative signal in subtype-specific data relates to papillary histology, and a causal pathway to clear-cell tumors remains undemonstrated, with shared metabolic and inflammatory factors offering a more parsimonious explanation. This case underscores the importance of thorough cross-sectional evaluation in patients with renal stones, particularly when ultrasound findings are equivocal, so that a coexisting solid renal mass is not overlooked.

It also supports nephron-sparing surgery with simultaneous stone removal as a feasible and oncologically sound strategy in suitably selected exophytic tumors. Further research integrating stone composition, metabolic profiling, and microbiome analysis is needed to clarify whether and how the lithogenic environment contributes to parenchymal carcinogenesis.
